# Quantitative Susceptibility Mapping and Amide Proton Transfer-Chemical Exchange Saturation Transfer for the Evaluation of Intracerebral Hemorrhage Model

**DOI:** 10.3390/ijms24076627

**Published:** 2023-04-01

**Authors:** Reika Sawaya, Junpei Ueda, Shigeyoshi Saito

**Affiliations:** 1 Department of Medical Physics and Engineering, Division of Health Sciences, Osaka University Graduate School of Medicine, Suita 560-0871, Osaka, Japan; u010443b@ecs.osaka-u.ac.jp (R.S.); uedaj@sahs.med.osaka-u.ac.jp (J.U.); 2 Department of Advanced Medical Technologies, National Cardiovascular and Cerebral Research Center, Suita 564-8565, Osaka, Japan

**Keywords:** preclinical 7T-MRI, amide proton transfer, chemical exchange saturation transfer, quantitative susceptibility mapping, intracerebral hemorrhage

## Abstract

This study aimed to evaluate an intracerebral hemorrhage (ICH) model using quantitative susceptibility mapping (QSM) and chemical exchange saturation transfer (CEST) with preclinical 7T-magnetic resonance imaging (MRI) and determine the potential of amide proton transfer-CEST (APT-CEST) for use as a biomarker for the early detection of ICH. Six Wistar male rats underwent MRI, and another six underwent histopathological examinations on postoperative days 0, 3, and 7. The ICH model was created by injecting bacterial collagenase into the right hemisphere of the brain. QSM and APT-CEST MRI were performed using horizontal 7T-MRI. Histological studies were performed to observe ICH and detect iron deposition at the ICH site. T_2_-weighted images (T_2_WI) revealed signal changes associated with hemoglobin degeneration in red blood cells, indicating acute-phase hemorrhage on day 0, late-subacute-phase hemorrhage on day 3, and chronic-phase hemorrhage on day 7. The susceptibility alterations in each phase were detected using QSM. QSM and Berlin blue staining revealed hemosiderin deposition in the chronic phase. APT-CEST revealed high magnetization transfer ratios in the acute phase. Abundant mobile proteins and peptides were observed in early ICH, which were subsequently diluted. APT-CEST imaging may be a reliable noninvasive biomarker for the early diagnosis of ICH.

## 1. Introduction

### 1.1. Intracerebral Hemorrhage

Intracerebral hemorrhage (ICH) accounts for 10–15% of all strokes and is associated with high morbidity and mortality rates [1,2]. The predictors of 30-day mortality in patients with acute ICH include the size, expansion, and location of the hematoma [3,4]. Therefore, the identification and quantification of the bleeding site are critical for diagnosing and predicting the prognosis of ICH. During the degradation of hemorrhage, diamagnetic hemoglobin is converted into paramagnetic oxyhemoglobin, deoxyhemoglobin, methemoglobin, and hemosiderin over time [5,6]. Accurate identification of the bleeding stage and quantitative tracking of the iron content can provide valuable insights into disease progression and treatment efficacy.

### 1.2. MR Imaging Technique for ICH

Computed tomography (CT) is considered the gold standard for the diagnosis of ICH; however, magnetic resonance imaging (MRI) is being used increasingly in the follow-up of ICH. Although T_1_-weighted images (T_1_WI) and T_2_-weighted images (T_2_WI) are used to evaluate ICH, conventional T_1_WI and T_2_WI are not sensitive to hemorrhage in the hyperacute stage [7] and are not quantitative. Gradient echo (GRE) MRI sequences are sensitive to local magnetic inhomogeneities, and T_2_*-weighted images show accuracy similar to that of CT for the detection of intraparenchymal hemorrhage. Moreover, its ability to detect chronic hemorrhage is superior to that of CT [8]. However, quantitative measurement of the volume of the hemorrhage remains challenging, as the signal intensity in T_2_^*^-weighted images is affected by several parameters, such as the echo time (TE), the magnetic field strength, and the voxel size [8].

### 1.3. Quantitative Susceptibility Mapping

Susceptibility-weighted imaging (SWI) and quantitative susceptibility mapping (QSM) are imaging techniques that measure and display magnetic susceptibility. SWI produces a qualitative image; in contrast, QSM produces a quantitative image independent of the imaging parameters. Moreover, unlike SWI, blooming artifacts and phase wrapping are not observed with QSM, which enables reliable monitoring of hemorrhage [9,10]. QSM consists of the processing phase and magnitude images reconstructed from real and imaginary images acquired using three-dimensional (3D) multi-echo GRE. A map of field inhomogeneities is estimated by removing the phase fold from the phase data. Subsequently, contributions from the background fields are removed, and a local magnetic field map generated by susceptibility sources within the region of interest (ROI) is extracted. QSM can be performed using dipole field inversion operations [11]. Several studies have used SWI and QSM for the evaluation of ICH [6,9,12], and a good correlation has been observed between the hemorrhage volumes calculated using CT and QSM [4].

### 1.4. Chemical Exchange Saturation Transfer

Chemical exchange saturation transfer (CEST) imaging is a noninvasive molecular imaging technique that measures low-concentration endogenous metabolites [13]. Protons, such as amino protons (-NH_2_), amide protons (-NH), and hydroxyl protons (-OH), are selectively saturated by a magnetization transfer (MT) pulse in CEST imaging. Subsequently, the magnetic saturation is transferred between the excited metabolite protons and non-excited water protons through a chemical exchange. Notably, repeating this process indirectly decreases the signal. The number of low-concentration solutes can be determined by measuring this attenuation, enabling the distribution of metabolites in the body to be displayed on a color map.

CEST imaging has been performed on various metabolites, such as amide protons [14], lactate [15], creatine [16], and glycosaminoglycans [17]. Amide proton transfer (APT)-CEST imaging can detect an increase in the concentration of amide protons of mobile proteins and peptides in brain tumors [18]. APT-CEST can be used to evaluate the tumor grade, predict treatment response and differentiate radiation necrosis from tumor recurrence. Hyperacute hemorrhage is also characterized by the presence of abundant mobile proteins and peptides, and it was recently reported that APT-CEST could accurately detect hyperacute ICH and distinguish it from cerebral ischemia [19]. However, no studies have made use of both QSM and APT-CEST to longitudinally evaluate an ICH model.

This study aimed to evaluate an ICH rat model using QSM and APT-CEST with preclinical 7T-MRI and determine the potential of APT-CEST to act as a biomarker for the early detection of ICH.

## 2. Results

### 2.1. ICH Area

**Figure 1:**
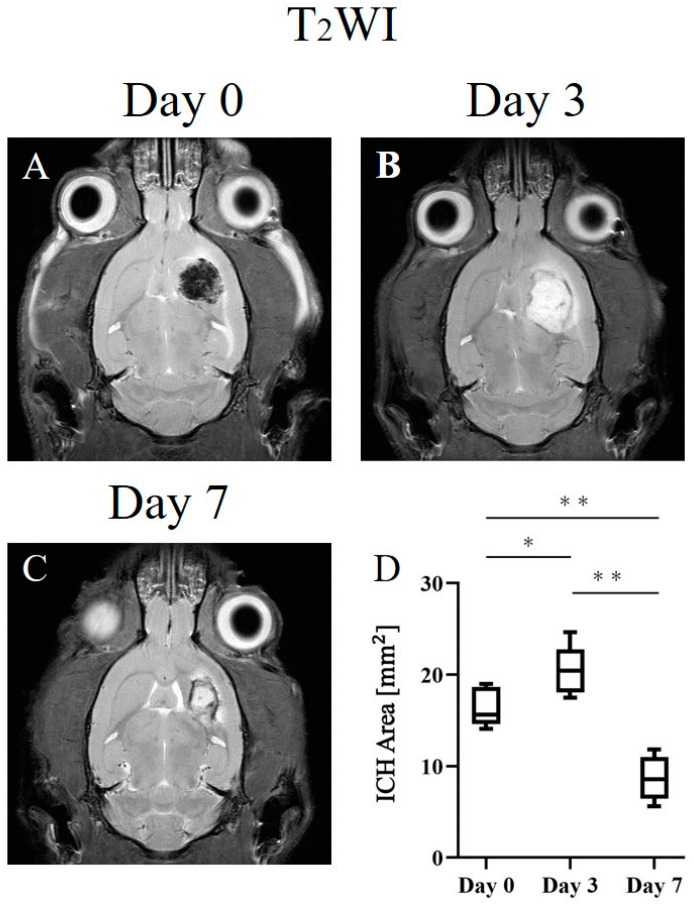
Representative T_2_W images of the intracranial hemorrhage (ICH) rat model on postoperative days 0 (**A**), 3 (**B**), and 7 (**C**). (**D**) The graph depicts the longitudinal changes in the ICH area. * *p* < 0.05; ** *p* < 0.01.

The hemorrhage caused by the injection of collagenase showed changes in composition and size as time progressed. Figure 1 depicts representative images acquired using T_2_WI at various stages and a graph showing the change in the size of the ICH area. T_2_WI acquired on day 0 revealed that compared with the normal area, the hemorrhagic area was hypointense (Figure 1A), whereas it was hyperintense on day 3 (Figure 1B). On day 7, T_2_WI revealed hypointensity at the limbus and hyperintensity at the center (Figure 1C). The ICH area increased significantly on day 3. In contrast, compared with that on days 0 and 3, the ICH area decreased significantly on day 7 (Figure 1D; 16.3 ± 2.0 mm^2^ (day 0) vs. 20.6 ± 2.6 mm^2^ (day 3), *p* < 0.05; 20.6 ± 2.6 mm^2^ (day 3) vs. 8.7 ± 2.5 mm^2^ (day 7), *p* < 0.01; 16.3 ± 2.0 mm^2^ (day 0) vs. 8.7 ± 2.5 mm^2^ (day 7), *p* < 0.01).

### 2.2. Quantitative Susceptibility Mapping

**Figure 2:**
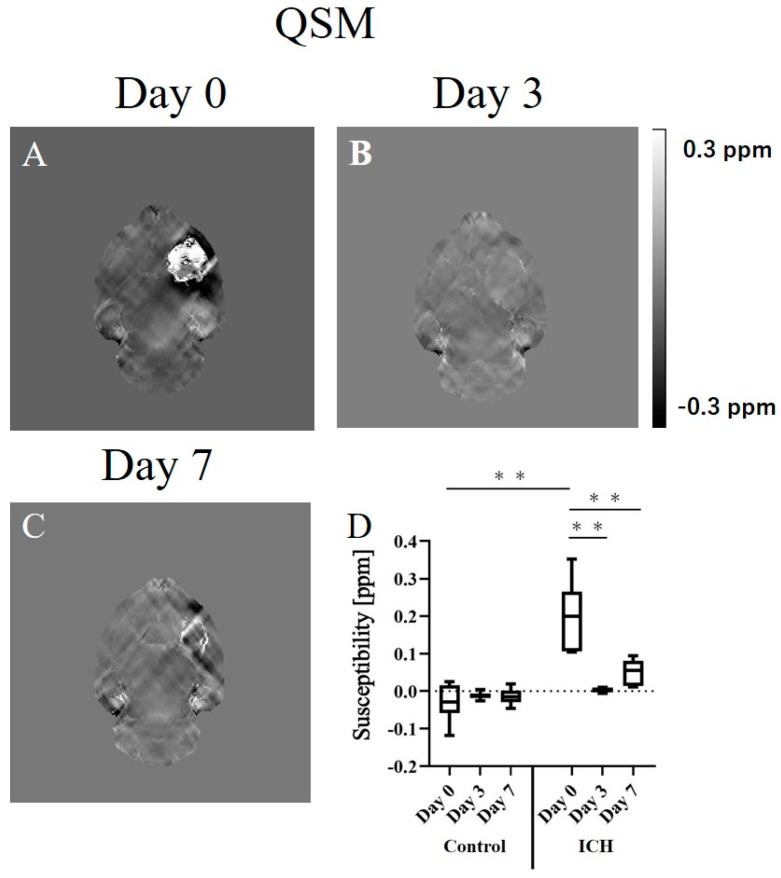
Representative quantitative susceptibility mapping (QSM) images of the intracranial hemorrhage (ICH) rat model on postoperative days 0 (**A**), 3 (**B**), and 7 (**C**). (**D**) The graph depicts the longitudinal changes in the susceptibility of the hemorrhage and normal area. ** *p* < 0.01.

Hemorrhage sites exhibited changes in susceptibility due to compositional variations over time. Figure 2 depicts representative images acquired using QSM at various stages and a graph showing the susceptibility values of the cerebral hemorrhage and normal areas. QSM images acquired on day 0 revealed the susceptibility of the hemorrhagic area was higher than that of the normal area (Figure 2A). On day 3, the susceptibility was almost equal to that of the normal area (Figure 2B). On day 7, high susceptibility was observed at the limbus of the hemorrhagic area (Figure 2C). Figure 2D depicts graphs showing the longitudinal changes in the susceptibility of the hemorrhagic and normal areas. The susceptibility of the hemorrhagic area was significantly higher than that of the normal area on day 0; however, no significant difference was observed between the hemorrhagic and normal areas on days 3 and 7. In addition, the longitudinal changes in the susceptibility of cerebral hemorrhage were significantly higher only on day 0; no significant difference was observed between days 3 and 7. The susceptibility values obtained using QSM were as follows: day 0 (control), −0.030 ± 0.050 ppm; day 3, −0.012 ± 0.010 ppm; day 7, −0.014 ± 0.022 ppm; ICH: day 0, 0.200 ± 0.090 ppm (vs. control day 0, *p* < 0.01); day 3, 0.004 ± 0.007 ppm (vs. ICH day 0, *p* < 0.01); day 7, 0.051 ± 0.035 ppm (vs. ICH day 0, *p* < 0.01).

### 2.3. APT-CEST Imaging

**Figure 3:**
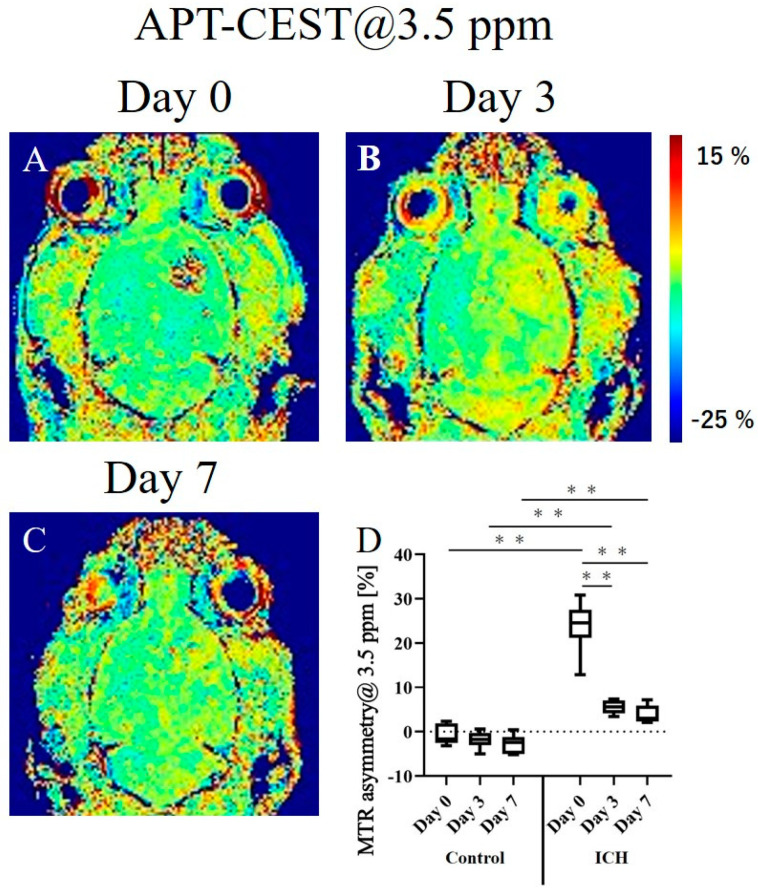
Representative MTR asymmetry maps of the intracranial hemorrhage (ICH) rat model obtained using APT-CEST imaging on postoperative days 0 (**A**), 3 (**B**), and 7 (**C**). (**D**) The graph depicts the longitudinal changes in MTR asymmetry value at 1.8 ppm of the hemorrhage site and the normal site. ** *p* < 0.01.

In APT-CEST, the hemorrhage site showed a signature CEST effect. In addition, the MTR values varied over time. Figure 3 depicts representative images acquired using CEST imaging at various stages and a graph showing the MTR values of the hemorrhagic and normal areas. The MTR asymmetry maps were created at 3.5 ppm. The MTR asymmetry map revealed that the MTR values of the hemorrhagic area were higher than those of the normal area on day 0 (Figure 3A). Thereafter, the MTR values of the hemorrhagic area tended to decrease gradually (Figure 3B,C). Figure 3D depicts graphs showing the longitudinal change in the MTR values of the hemorrhagic and normal areas. The values were almost the same at all time points and showed no changes in the normal area. In contrast, the values were very high on day 0 and decreased thereafter in the hemorrhagic area. The MTR values of the hemorrhagic area were significantly higher than those of the normal area at all time points on days 0, 3, and 7. The longitudinal change in the MTR values of the cerebral hemorrhage area was significantly higher only on day 0, and no significant difference was observed between days 3 and 7. The MTR values obtained using APT-CEST imaging were as follows: day 0 (control), −0.8 ± 2.3% (vs. ICH on day 0, *p* < 0.01); day 3, −1.8 ± 1.9% (vs. ICH on day 3, *p* < 0.01); day 7, −2.8 ± 2.1% (vs. ICH on day 7, *p* < 0.01); ICH: day 0, 23.9 ± 5.9%; day 3, 5.5 ± 1.5% (vs. ICH on day 0, *p* < 0.01); day 7, 3.8 ± 2.1% (vs. ICH on day 0, *p* < 0.01).

### 2.4. Histological Studies

**Figure 4:**
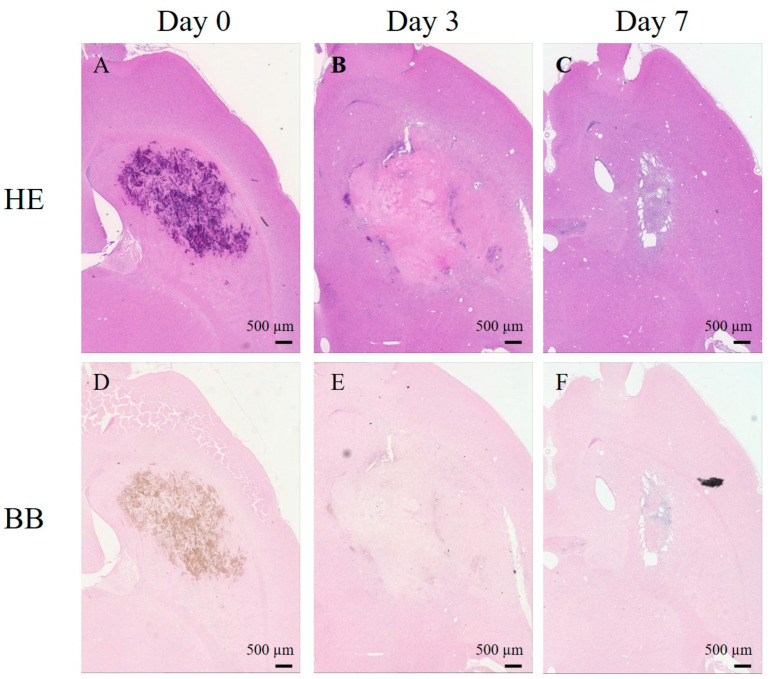
Histopathological changes in the intracranial hemorrhage area at various stages. (**A**–**C**) Hematoxylin and eosin (HE) staining. (**D**–**F**) Berlin blue (BB) staining.

HE and BB staining confirmed that the hematoma was certainly induced by collagenase injection and that it changed in composition and size over time. Figure 4 depicts histological sections of the ICH area at various stages. HE staining confirmed the formation of a hematoma, and as observed on the MRI images, the hemorrhagic area was enlarged on day 3, which subsequently reduced on day 7 (Figure 4A–C). BB staining revealed partially blue-stained areas on day 7 (Figure 4D–F).

## 3. Discussion

This is the first study to longitudinally observe an ICH model using QSM and APT-CEST imaging. T_2_WI revealed signal changes associated with the degeneration of hemoglobin in the red blood cells, indicating acute-phase hemorrhage on day 0, late-subacute-phase hemorrhage on day 3, and chronic-phase hemorrhage on day 7. The susceptibility alterations in each phase were detected using QSM. QSM and BB staining revealed hemosiderin deposition during the chronic phase. APT-CEST imaging revealed high MTR values in the acute phase. Early ICH was proven to be rich in mobile proteins and peptides, which were subsequently diluted.

### 3.1. ICH Progression and Staging

T_2_WI revealed a low signal on day 0, a high signal on day 3, a low signal at the limbus, and a high signal at the center on day 7 at the hemorrhage site (Figure 1). ICH is classified into five stages based on hemoglobin degeneration in the blood cells and its intra- and extracellular localization: hyperacute, acute, early subacute, late subacute, and chronic [20]. The following variations in hemoglobin and T_2_ values have been reported at various stages of ICH in rats: the hyperacute phase, 0–6 h (intracellular oxyhemoglobin, long T_2_); the acute phase (intracellular deoxyhemoglobin, short T_2_); the early subacute phase, 24–72 h (intracellular methemoglobin, short T_2_); the late subacute phase (extracellular methemoglobin, long T_2_); the chronic phase, 7 days (ferritin and hemosiderin, short T_2_) [5,21]. Previous studies have shown that the dephasing of proton spins as water moves into the erythrocytes, which contain paramagnetic deoxyhemoglobin and methemoglobin, resulting in low intensity during the early stages of hemorrhage. Methemoglobin is released during erythrocyte lysis, and it appears isointense or hyperintense on T_2_WI. Furthermore, the surrounding tissue shows a low intensity, as macrophages ingest extracellular iron to form hemosiderin [20,21,22]. The results of the present experiment were in complete agreement with those of a previous study. Thus, T_2_WI acquired in the present study depicted the characteristics of the acute phase of ICH on day 0, the late subacute phase on day 3, and the chronic phase on day 7. BB staining revealed blue staining on day 7 (Figure 4). BB stains hemosiderin, a trivalent iron ion [23]; thus, BB staining indicated the presence of hemosiderin during the chronic phase on day 7. The speed of ICH progression in the brain differs between humans and rats. Since hematomas are largely degraded by inflammatory cells that must migrate from the periphery, the rate of cell migration may affect the rate of hematoma resolution [20]. Thus, the rate of hematoma progression is faster in rats with smaller hematomas than that in other large animals.

### 3.2. ICH Area

In cerebral hemorrhage, the hematoma often enlarges or rebleeds after its occurrence, and shows a rapid increase with edema. Subsequently, the hematoma gradually disappears, resulting in the formation of a cavity caused by the destruction of brain tissue [3,24,25]. The collagenase model mimics this hematoma expansion. In the present study, the size of the cerebral hemorrhage followed a trajectory similar to those reported in previous studies. The areas showing cerebral hemorrhage were almost the same on T_2_WI, QSM, and APT-CEST. A similar trend was also observed in HE staining, wherein an enlargement of the damaged area of the cells was observed on day 3 and a decrease with cell loss was observed on day 7 (Figure 4). Determining the size of the hematoma is important for prognostic measurements in ICH, and it is suggested that QSM and APT-CEST can be useful tools for this purpose.

### 3.3. Quantitative Susceptibility Mapping

The QSM revealed the highest susceptibility on day 0, which decreased on day 3. On day 7, the susceptibility was higher at the site of the hemosiderin rings observed on T_2_WI. A significant increase in the susceptibility compared with that of the normal site was shown only on day 0 (Figure 2). A previous study that observed ICH using QSM in human patients [6] revealed that the susceptibility is highest during the acute phase. Thereafter, the susceptibility decreased from the acute to late subacute phases and increased during the chronic phase. The results of the present experiment showed similar trends. Deoxyhemoglobin showed high susceptibility on day 0, methemoglobin showed low susceptibility on day 3, and hemosiderin showed high susceptibility on day 7. Thus, QSM showed signal variations that were specific to each stage.

### 3.4. APT-CEST Imaging

APT-CEST imaging is based on the chemical exchange of bulk water with a selectively saturated proton of an amide group (-NH) [26]. Therefore, the MTR asymmetry map created at 3.5 ppm, which was the resonance frequency of the amide group, enabled us to observe the concentration and distribution of mobile proteins and peptides in vivo. As shown in Figure 3, the MTR values of the hemorrhagic area were significantly higher than those of the normal area at all time points on days 0, 3, and 7. Changes over time at the hemorrhage sites were significantly higher on day 0 and decreased thereafter, with no significant differences observed between days 3 and 7. Previous studies have reported that the APT signals are consistently higher in hyperacute ICH 12 h after the injection of collagenase than those in contralateral brain tissue [19]. It was also reported that APT-CEST is successful in detecting the hyperacute, acute, and subacute phases of ICH [27]. The results of the present study are consistent with those of previous studies. The present study revealed the abundance of endogenous mobile proteins and peptides in acute hemorrhage. Mobile proteins and peptides are introduced into hematomas via blood agents, such as hemoglobin-rich red blood cells, white blood cells, platelet clumps, and protein-rich serum collections [27]. Subsequently, the protein content decreases in the subacute and chronic phases but remains significantly higher in the cerebral hemorrhage area than that in the normal area. However, there was no marked difference in the MTR values on days 3 and 7, making it difficult to distinguish between the subacute and chronic phases based on images. In contrast, QSM images reflected the differences in susceptibility due to hemoglobin degeneration at each phase of the disease. The rate of proton exchange is highly dependent on the pH in CEST imaging [28,29]. ICH increases the pH to be higher than that in normal areas, which may have influenced the elevated MTR values [19,27]. As a point of concern, the SD of the MTR value was very large on day 0, possibly due to the signal from collagenase itself.

In this study, experiments using ultra-high field 7T-MRI on rats have revealed the potential of APT-CEST as a biomarker for the early diagnosis of ICH. However, its clinical application in humans may still be challenging due to the longer scan time compared to other imaging methods and the lower specificity of the CEST effect in low-field MRI [30,31]. Therefore, there is a need to develop faster CEST methods and more specific analysis techniques in the future to improve its usefulness [32,33]. This could make APT-CEST imaging useful not only for the evaluation of brain tumors, but also for the detection of ICH and differentiation between ICH and ischemia.

### 3.5. Limitations

This study had several limitations. First, the sample size was small, as we only used six animals to obtain data. The time points were also limited (days 0, 3, and 7). Hematomas break down more rapidly in rats than in humans due to differences in the rate of erythrocyte degradation and the size of the hematoma [20,34]. Therefore, it was necessary to set time points at shorter intervals to observe the changes in the MRI signal associated with alterations in the composition of the red blood cells. Further information regarding the early stages of hemorrhage is necessary, particularly in APT-CEST, as the signal dropped rapidly from day 0 to day 3.

Second, only T_2_W, QSM, and APT-CEST imaging were performed in this study. It is necessary to make comparisons with various imaging methods, such as T_1_W imaging, T_2_*W imaging, SWI, and DWI, to closely investigate the peculiarities and advantages of each imaging method.

Third, an ultra-high-field 7T-MRI with a large B_1_ inhomogeneity was used in this study. However, a correction for B_1_ inhomogeneity was not made for CEST imaging. B_1_ inhomogeneity affects the signal-to-noise ratio of the CEST signal and the image contrast, and the contrast may be inhomogeneous in the entire FOV without B_1_ correction [35,36]. Therefore, B_1_ correction could result in a more accurate CEST image.

## 4. Materials and Methods

### 4.1. Animal Preparation

All animal procedures were approved by the Research Ethics Committee of Osaka University (R02-05-0). All experimental procedures involving animals and their care adhered to the Osaka University Guidelines for Animal Experimentation. Male Wistar rats were purchased from SLC Japan (Hamamatsu, Japan). Six rats (aged 10–11 weeks, 220–280 g) underwent imaging, and another six (aged 10 weeks, 220–250 g) underwent histopathological examinations. All rats were housed in a controlled vivarium environment (24 °C; 12:12 h light:dark cycle) and fed a standard pellet diet and water ad libitum.

Collagenase injections mimic the effects of cerebral hemorrhage by destroying the vascular tissue [3,25,37]. Therefore, the ICH model was created by injecting bacterial collagenase directly into the right hemisphere of the brain as described below. The rats were anesthetized with isoflurane (Wako Pure Chemical Industries, Ltd., Osaka, Japan) and immobilized using a stereotactic frame (Stereotaxic Instruments for Rats; Narishige Scientific Instrument Lab., Tokyo, Japan). A scalpel was used to incise the scalp and expose the cranium. An electric drill was used to create a hole with a size of 3 mm to the right of the bregma. Collagenase (type IV; Sigma-Aldrich Japan, Tokyo, Japan) was dissolved in 0.5 μL saline and injected into the striatum at a depth of 4 mm from the cranial surface using a microinjector over the course of 5 min. The needle was retracted slowly after the injection, and the wound was closed.

The rats underwent MRI examinations on postoperative days 0, 3, and 7. For the pathological examination, two rats were sacrificed on postoperative days 0, 3, and 7, and their brains were harvested. The rats were anesthetized with isoflurane (3.0% for induction and 1.5% for maintenance) during all experiments, and their body temperatures were maintained at 36.5 °C with regulated water flow. In addition, their respiration was continuously monitored using a physiological monitoring system (SA Instruments, Inc., Stony Brook, NY, USA).

### 4.2. MRI Examinations

All MRI examinations were performed on a horizontal 7T-MRI system (PharmaScan 70/16 US, Bruker BioSpin, Ettlingen, Germany) equipped with a transmit/receive volume-radio-frequency (RF) coil with a diameter of 40 mm. Coronal three-dimensional (3D) T_2_W and QSM images were acquired in the same imaging fields, and CEST data of the cross-section with the largest intracerebral hemorrhage were acquired. T_2_WI were acquired using turbo rapid acquisition with a relaxation enhancement (Turbo RARE) sequence with the following parameters: repetition time (TR)/TE = 3000/33 ms; number of slices = 10; RARE factor = 8; field of view = 36.0 × 36.0 mm^2^; matrix size = 300 × 300; slice thickness = 1.0 mm; number of averages = 2.

The QSM sequence parameters were as follows: 3D multi-echo GRE; TR/TE = 100/4 ms; flip angle = 15°; echo images = 5; field of view = 36.0 × 36.0 × 10.0 mm^3^; matrix size = 300 × 300 × 10; number of averages = 2.

The CEST data were acquired using MT rapid acquisition with a refocused echo (RARE) sequence. The sequence parameters were as follows: TR/TE = 2200/33 ms; field of view = 36.0 × 36.0 mm^2^; slice thickness = 1 mm; matrix size = 128 × 128; number of averages = 1; in-plane resolution = 281 × 281 µm^2^. The MT pulse parameters were as follows: shape of the continuous-wave saturation pulse = block pulse; length = 100 ms; number of pulses = 20; interpulse delay = 0.01 ms; bandwidth = 12.8 Hz; B_1_ amplitude = 0.5 µT; flip angle = 766°. The Z-spectrum was obtained from CEST images with varying saturation frequencies ranging from −5.0 ppm to +5.0 ppm (0.5 ppm per step; 21 images) and one S_0_ image (without an MT pulse). Point-by-point B_0_ correction was performed using water saturation shift referencing (WASSR) [38]. The B_1_ amplitude was set to 0.3 µT, and saturation offsets were set from −1.0 ppm to +1.0 ppm (0.1 ppm per step; 21 images) to obtain the WASSR data. The total acquisition time per animal was approximately 40 min.

### 4.3. Data Processing

An ROI was placed over the entire cerebral hemorrhage on T_2_WI to measure the size of the cerebral hemorrhage (mm^2^) on postoperative days 0, 3, and 7. QSM and CEST imaging calculated the susceptibility values (ppm) and MTR values (%), respectively, as quantitative values. The same ROI as that set on T_2_WI was fixed on the QSM and CEST images to calculate the susceptibility and MTR values at each time point. The ROIs were set on the left hemisphere of the brain to compare the quantitative values of the ICH area with those of the normal area. The size of the ICH area and the susceptibility and MTR values were obtained using ImageJ software (National Institutes of Health, Bethesda, MD, USA).

All processing and data analyses pertaining to QSM and CEST imaging were performed using in-house scripts written on MATLAB (2021, MathWorks, Inc., Natick, MA, USA). QSM was performed using the morphology-enabled dipole inversion (MEDI) method [39,40]. MTR asymmetry (MTR value) was calculated using the following equation: MTR_asym_ (%) = S_(−αppm)_ − S_(+αppm)_ / S_0_. The APT-CEST signal was defined as an MTR asymmetry of +3.5 ppm, and MTR asymmetry maps were created at 3.5 ppm. Since some pixels had missing values due to calculation errors, the maximum MTR value in the ROI was calculated and compared instead of the average value.

### 4.4. Histopathology

The rats were sacrificed (n = 2 per time point) subsequent to undergoing MRI on postoperative days 0, 3, and 7, and their brains were harvested. The histological evaluation of ICH was performed using hematoxylin and eosin (HE) staining, and the presence of iron deposition at the site of ICH was detected using Berlin blue (BB) staining. The brain tissue was fixed in 4% phosphate-buffered formaldehyde, embedded in paraffin wax and sectioned at 5 µm. The sections were degreased with xylene and rehydrated with a series of ethanol−water washes. After washing with distilled water, the sections were incubated with hematoxylin for 4 min and washed with purified water for 15 min. The sections were then incubated for 2 min with eosin, rinsed with purified water, rehydrated with a series of ethanol−water washes and degreased with xylene.

BB staining was performed as described below. After rinsing with distilled water, the sections were incubated with a BB staining solution for 30 min. The staining solution was prepared by mixing 50 mL each of a 2% potassium ferrocyanide solution (Sigma-Aldrich Japan, Tokyo, Japan) and 1% hydrochloric acid (Sigma-Aldrich Japan, Tokyo, Japan) in equal volumes. The sections were then rinsed in running water, incubated in a Cologne echolate solution (Muto Pure Chemicals Co., Tokyo, Japan) for 5 min and dehydrated with alcohol. Subsequently, the ethanol in the tissues was replaced with xylene, and the brain tissue was examined under a fluorescence microscope (BZ-X810; Keyence, Osaka, Japan).

### 4.5. Statistical Analysis

The size of the ICH area (mm^2^) and the susceptibility (ppm) and MTR asymmetry values (%) are presented as mean ± standard deviation (SD). One-way ANOVA with Tukey’s multiple-comparison tests was performed using Prism 8 (version 8, GraphPad Software, San Diego, CA, USA) to compare the differences in areas, susceptibility, and MTR asymmetry values among the groups. The differences were considered statistically significant at a *p*-value of < 0.05. The notations for significant differences in the graphs of the results are as follows: * *p* < 0.05; ** *p* < 0.01.

## 5. Conclusions

We longitudinally observed ICH using QSM and APT-CEST in a collagenase-injected rat model. QSM could detect a change in the susceptibility of hematomas, suggesting that it is useful for estimating the disease stage. Furthermore, APT-CEST imaging may be a reliable noninvasive biomarker for the early diagnosis of ICH.

## Data Availability

The data presented in this study are available on request from the corresponding author.

## References

[1] Ikram M.A., Wieberdink R.G., Koudstaal P.J. (2012). International epidemiology of intracerebral hemorrhage. Curr. Atheroscler Rep..

[2] Sun H., Klahr A.C., Kate M., Gioia L.C., Emery D.J., Butcher K.S., Wilman A.H. (2018). Quantitative Susceptibility Mapping for Following Intracranial Hemorrhage. Radiology.

[3] MacLellan C.L., Silasi G., Auriat A.M., Colbourne F. (2010). Rodent models of intracerebral hemorrhage. Stroke.

[4] Zhang Y., Wei H., Sun Y., Cronin M.J., He N., Xu J., Zhou Y., Liu C. (2018). Quantitative susceptibility mapping (QSM) as a means to monitor cerebral hematoma treatment. J. Magn. Reson. Imaging.

[5] Bradley W.G. (1993). MR appearance of hemorrhage in the brain. Radiology.

[6] Chang S., Zhang J., Liu T., Tsiouris A.J., Shou J., Nguyen T., Leifer D., Wang Y., Kovanlikaya I. (2016). Quantitative Susceptibility Mapping of Intracerebral Hemorrhages at Various Stages. J. Magn. Reson. Imaging.

[7] Kidwell C.S., Wintermark M. (2008). Imaging of intracranial haemorrhage. Lancet Neurol..

[8] Wang S., Lou M., Liu T., Cui D., Chen X., Wang Y. (2013). Hematoma volume measurement in gradient echo MRI using quantitative susceptibility mapping. Stroke.

[9] De A., Sun H., Emery D.J., Butcher K.S., Wilman A.H. (2022). Quantitative susceptibility-weighted imaging in presence of strong susceptibility sources: Application to hemorrhage. Magn. Reson. Imaging.

[10] Lin F., Prince M.R., Spincemaille P., Wang Y. (2019). Patents on Quantitative Susceptibility Mapping (QSM) of Tissue Magnetism. Recent Pat. Biotechnol..

[11] Ruetten P.P.R., Gillard J.H., Graves M.J. (2019). Introduction to Quantitative Susceptibility Mapping and Susceptibility Weighted Imaging. Br. J. Radiol..

[12] Weerink L.B., Appelman A.P., Kloet R.W., Van der Hoorn A. (2022). Susceptibility-weighted imaging in intracranial hemorrhage: Not all bleeds are black. Br. J. Radiol..

[13] Ward K.M., Aletras A.H., Balaban R.S. (2000). A new class of contrast agents for MRI based on proton chemical exchange dependent saturation transfer (CEST). J. Magn. Reson..

[14] Tanoue M., Saito S., Takahashi Y., Araki R., Hashido T., Kioka H., Sakata Y., Yoshioka Y. (2019). Amide proton transfer imaging of glioblastoma, neuroblastoma, and breast cancer cells on a 11.7 T magnetic resonance imaging system. Magn. Reson. Imaging.

[15] Saito S., Takahashi Y., Ohki A., Shintani Y., Higuchi T. (2019). Early detection of elevated lactate levels in a mitochondrial disease model using chemical exchange saturation transfer (CEST) and magnetic resonance spectroscopy (MRS) with 7T MR imaging. Radiol. Phys. Technol..

[16] Sawaya R., Kuribayashi S., Ueda J., Saito S. (2022). Evaluating the Cisplatin Dose Dependence of Testicular Dysfunction Using Creatine Chemical Exchange Saturation Transfer Imaging. Diagnostics.

[17] Wada T., Togao O., Tokunaga C., Funatsu R., Yamashita Y., Kobayashi K., Nakamura Y., Honda H. (2017). Glycosaminoglycan chemical exchange saturation transfer in human lumbar intervertebral discs: Effect of saturation pulse and relationship with low back pain. J. Magn. Reson. Imaging.

[18] Onishi R., Sawaya R., Tsuji K., Arihara N., Ohki A., Ueda J., Hata J., Saito S. (2022). Evaluation of Temozolomide Treatment for Glioblastoma Using Amide Proton Transfer Imaging and Diffusion MRI. Cancers.

[19] Wang M., Hong X., Chang C.F., Li Q., Ma B., Zhang H., Xiang S., Heo H.Y., Zhang Y., Lee D.H. (2015). Simultaneous detection and separation of hyperacute intracerebral hemorrhage and cerebral ischemia using amide proton transfer MRI. Magn. Reson. Med..

[20] Del Bigio M.R., Yan H.J., Buist R., Peeling J. (1996). Experimental intracerebral hemorrhage in rats. Magnetic resonance imaging and histopathological correlates. Stroke.

[21] Belayev L., Obenaus A., Zhao W., Saul I., Busto R., Wu C., Vigdorchik A., Lin B., Ginsberg M.D. (2007). Experimental intracerebral hematoma in the rat: Characterization by sequential magnetic resonance imaging, behavior, and histopathology. Effect of albumin therapy. Brain Res..

[22] Liang J.J., Lei L., Zeng Y.P., Xiao Z.M. (2019). High signal-intensity abnormalities in susceptibility-weighted imaging for primary intracerebral hemorrhage. Int. J. Neurosci..

[23] Liu C., Li W., Tong K.A., Yeom K.W., Kuzminski S. (2015). Susceptibility-weighted imaging and quantitative susceptibility mapping in the brain. J. Magn. Reson. Imaging.

[24] Fujii Y., Tanaka R., Takeuchi S., Koike T., Minakawa T., Sasaki O. (1994). Hematoma enlargement in spontaneous intracerebral hemorrhage. J. Neurosurg..

[25] Keep R.F., Hua Y., Xi G. (2012). Intracerebral haemorrhage: Mechanisms of injury and therapeutic targets. Lancet Neurol..

[26] Zhou J., Payen J.F., Wilson D.A., Traystman R.J., van Zijl P.C. (2003). Using the amide proton signals of intracellular proteins and peptides to detect pH effects in MRI. Nat. Med..

[27] Ma X., Bai Y., Lin Y., Hong X., Liu T., Ma L., Haacke E.M., Zhou J., Wang J., Wang M. (2017). Amide proton transfer magnetic resonance imaging in detecting intracranial hemorrhage at different stages: A comparative study with susceptibility weighted imaging. Sci. Rep..

[28] Jin T., Wang P., Zong X., Kim S.G. (2013). MR imaging of the amide-proton transfer effect and the pH-insensitive nuclear overhauser effect at 9.4 T. Magn. Reson. Med..

[29] Zhou J., Heo H.Y., Knutsson L., van Zijl P.C.M., Jiang S. (2019). APT-weighted MRI: Techniques, current neuro applications, and challenging issues. J. Magn. Reson. Imaging.

[30] Dula A.N., Smith S.A., Gore J.C. (2013). Application of chemical exchange saturation transfer (CEST) MRI for endogenous contrast at 7 Tesla. J. Neuroimaging.

[31] Perlman O., Farrar C.T., Heo H.Y. (2022). MR fingerprinting for semisolid magnetization transfer and chemical exchange saturation transfer quantification. NMR Biomed..

[32] Jones K.M., Pollard A.C., Pagel M.D. (2018). Clinical applications of chemical exchange saturation transfer (CEST) MRI. J. Magn. Reson. Imaging.

[33] Zaiss M., Xu J., Goerke S., Khan I.S., Singer R.J., Gore J.C., Gochberg D.F., Bachert P. (2014). Inverse Z-spectrum analysis for spillover-, MT-, and T1 -corrected steady-state pulsed CEST-MRI--application to pH-weighted MRI of acute stroke. NMR Biomed..

[34] An D., Park J., Shin J.I., Kim H.J., Jung D.I., Kang J.H., Kim G., Chang D.W., Sur J.H., Yang M.P. (2015). Temporal Evolution of MRI Characteristics in Dogs with Collagenase-Induced Intracerebral Hemorrhage. Comp. Med..

[35] Rerich E., Zaiss M., Korzowski A., Ladd M.E., Bachert P. (2015). Relaxation-compensated CEST-MRI at 7 T for mapping of creatine content and pH--preliminary application in human muscle tissue in vivo. NMR Biomed..

[36] Windschuh J., Zaiss M., Meissner J.E., Paech D., Radbruch A., Ladd M.E., Bachert P. (2015). Correction of B1-inhomogeneities for relaxation-compensated CEST imaging at 7 T. NMR Biomed..

[37] Chang C.F., Massey J., Osherov A., Angenendt da Costa L.H., Sansing L.H. (2020). Bexarotene Enhances Macrophage Erythrophagocytosis and Hematoma Clearance in Experimental Intracerebral Hemorrhage. Stroke.

[38] Kim M., Gillen J., Landman B.A., Zhou J., van Zijl P.C. (2009). Water saturation shift referencing (WASSR) for chemical exchange saturation transfer (CEST) experiments. Magn. Reson. Med..

[39] Liu J., Liu T., de Rochefort L., Ledoux J., Khalidov I., Chen W., Tsiouris A.J., Wisnieff C., Spincemaille P., Prince M.R. (2012). Morphology enabled dipole inversion for quantitative susceptibility mapping using structural consistency between the magnitude image and the susceptibility map. Neuroimage.

[40] Liu T., Xu W., Spincemaille P., Avestimehr A.S., Wang Y. (2012). Accuracy of the morphology enabled dipole inversion (MEDI) algorithm for quantitative susceptibility mapping in MRI. IEEE Trans. Med. Imaging.

